# Aqua­(3-carboxybenzoato-κ*O* ^1^)(nitrato-κ*O*)(dipyrido[3,2-*a*:2′,3′-*c*]phenazine-κ^2^ *N* ^4^,*N* ^5^)copper(II)

**DOI:** 10.1107/S1600536808029449

**Published:** 2008-09-20

**Authors:** Yan An, Xiao-Feng Li, Li-Hua Dong, Yan-Sheng Yin

**Affiliations:** a Institute of Marine Materials Science and Engineering, Shanghai Maritime University, Shanghai 200135, People’s Republic of China

## Abstract

The title complex, [Cu(C_8_H_5_O_4_)(NO_3_)(C_18_H_10_N_4_)(H_2_O)], was synthesized by reacting Cu(NO_3_)_2_, isophthalic acid and dipyridophenazine under hydro­thermal conditions. The Cu^II^ ion is in a slightly distorted square-pyramidal coordination environment. In the crystal structure, inter­molecular O—H⋯O hydrogen bonds connect complex mol­ecules into chains along [001].

## Related literature

For related literature, see: Gupta *et al.* (1992 ▶); Han & Ma (2006 ▶); Han *et al.* (2007 ▶); Hartshorn & Barton (1992 ▶); He & Han (2006 ▶); Murphy *et al.* (1993 ▶).
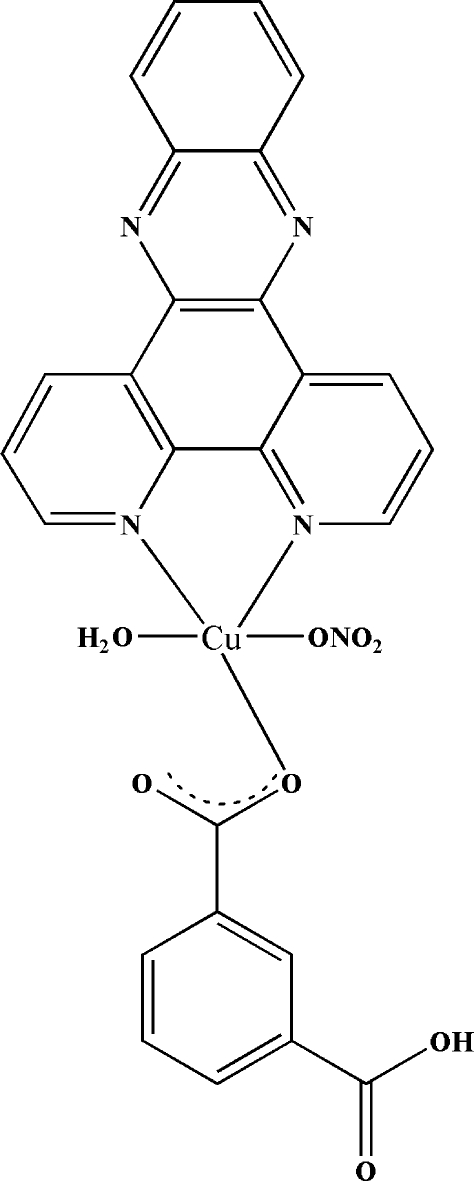


## Experimental

### 

#### Crystal data

[Cu(C_8_H_5_O_4_)(NO_3_)(C_18_H_10_N_4_)(H_2_O)]
*M* *_r_* = 590.99
Triclinic, 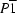

*a* = 7.8965 (17) Å
*b* = 11.295 (4) Å
*c* = 14.533 (6) Å
α = 112.73 (3)°
β = 90.94 (3)°
γ = 102.60 (2)°
*V* = 1159.4 (6) Å^3^
*Z* = 2
Mo *K*α radiation
μ = 1.01 mm^−1^
*T* = 293 (2) K
0.37 × 0.32 × 0.24 mm

#### Data collection

Bruker APEX area-detector diffractometer
Absorption correction: multi-scan (*SADABS*; Sheldrick, 1996 ▶) *T*_min_ = 0.707, *T*_max_ = 0.797
6320 measured reflections
5180 independent reflections
3353 reflections with *I* > 2σ(*I*)
*R*_int_ = 0.036

#### Refinement

*R*[*F* ^2^ > 2σ(*F* ^2^)] = 0.059
*wR*(*F* ^2^) = 0.198
*S* = 1.01
5180 reflections
367 parameters
39 restraints
H atoms treated by a mixture of independent and constrained refinement
Δρ_max_ = 0.60 e Å^−3^
Δρ_min_ = −0.92 e Å^−3^

### 

Data collection: *SMART* (Bruker, 2001 ▶); cell refinement: *SAINT* (Bruker, 2001 ▶); data reduction: *SAINT*; program(s) used to solve structure: *SHELXS97* (Sheldrick, 2008 ▶); program(s) used to refine structure: *SHELXL97* (Sheldrick, 2008 ▶); molecular graphics: *SHELXTL* (Sheldrick, 2008 ▶); software used to prepare material for publication: *SHELXTL*.

## Supplementary Material

Crystal structure: contains datablocks I, global. DOI: 10.1107/S1600536808029449/lh2688sup1.cif

Structure factors: contains datablocks I. DOI: 10.1107/S1600536808029449/lh2688Isup2.hkl

Additional supplementary materials:  crystallographic information; 3D view; checkCIF report

## Figures and Tables

**Table:** 

Cu1—O1*W*	1.952 (3)
Cu1—O3	1.960 (3)
Cu1—N1	2.000 (4)
Cu1—N2	2.001 (4)
Cu1—O7	2.284 (4)

**Table:** 

O1*W*—Cu1—O3	94.58 (14)
O1*W*—Cu1—N1	171.25 (17)
O3—Cu1—N1	89.65 (14)
O1*W*—Cu1—N2	92.03 (15)
O3—Cu1—N2	164.17 (16)
N1—Cu1—N2	82.02 (15)
O1*W*—Cu1—O7	97.54 (17)
O3—Cu1—O7	89.57 (16)
N1—Cu1—O7	90.14 (16)
N2—Cu1—O7	103.82 (17)

**Table 2:** Hydrogen-bond geometry (Å, °)

*D*—H⋯*A*	*D*—H	H⋯*A*	*D*⋯*A*	*D*—H⋯*A*
O1*W*—H1*WA*⋯O6^i^	0.841 (19)	2.08 (3)	2.856 (7)	153 (5)
O1*W*—H1*WA*⋯O5^i^	0.841 (19)	2.41 (3)	3.165 (6)	151 (6)
O1*W*—H1*WB*⋯O4	0.842 (19)	1.88 (4)	2.565 (5)	138 (6)
O2—H2*B*⋯O4^ii^	0.82	1.94	2.722 (4)	159

Symmetry codes: (i) 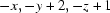; (ii) 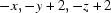.

**Fig. 1.**
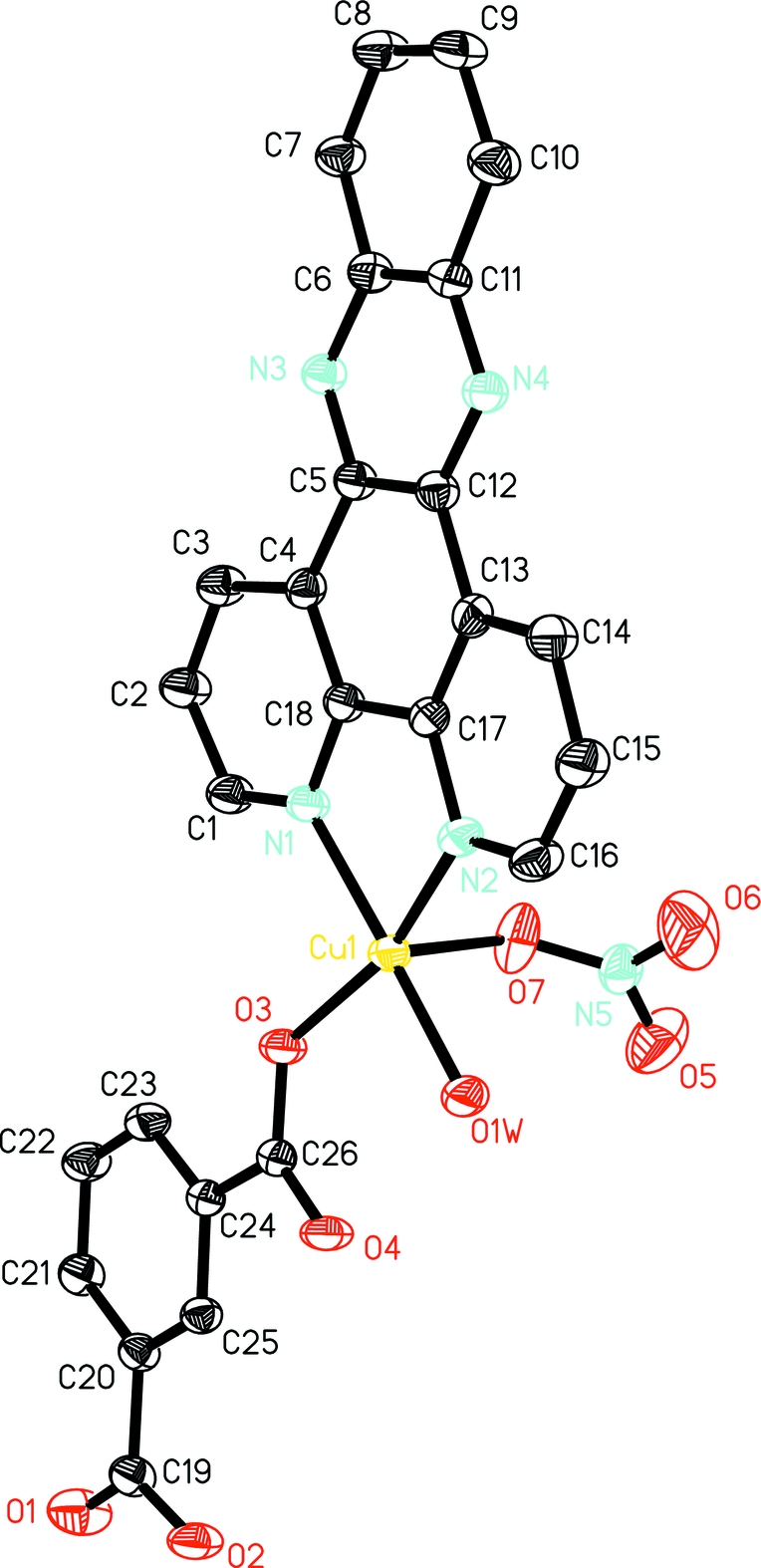
The molecular structure of (I). Displacement ellipsoids are drawn at the 30% probability level. H atoms have been omitted for clarity.

## supplementary crystallographic information

### Comment

Dipyridophenazine derivatives can be used as molecular light switches (Hartshorn & Barton, 1992) for the study of fast electron transfer through DNA (Murphy *et al.*, 1993). A dipyridophenazine ruthenium(II) complex has been found to be a good cleavage agent with high affinity for DNA (Gupta *et al.*, 1992). Recently, some examples of dinuclear copper(II) complexes of dipyridophenazine or isophthalate have been reported (He & Han, 2006; Han & Ma, 2006; Han *et al.*, 2007). The synthesis and crystal structure of a mononuclear copper(II) complex with a dipyridophenazine and a hydrogenisophthalato ligand is presented herein.

The title complex (I) (Fig. 1) is formed by one dipyridophenazine ligand, one NO_3_ ligand, one aqua ligand and one hydrogenisophthalato ligand coordinated to a Cu^II^ atom by three oxygen atoms and two nitrogen atoms in a slightly distorted square-pyramidal geometry. In the crystal structure, the mononuclear complex molecules are linked *via* intermolecular O—H···O hydrogen bonds (Table 2) forming one-dimensional chains along [001].

### Experimental

A mixture of Cu(NO_3_)_2_.2H_2_O (0.5 mmol, 0.120 g), isophthalic acid (0.5 mmol, 0.084 g), dipyridophenazine (0.5 mmol, 0.141 g) and water (10 ml) was mixed in a 23 ml Teflon reactor, which was heated at 453 K for six days and then cooled to room temperature at a rate of 5 K h^-1^. Yield: 58%. CH&N analysis for C_26_H_17_N_5_O_8_Cu (found/calc): C, 53.05 (52.84), H, 2.94 (2.90), N, 11.96% (11.85%).

### Refinement

H atoms were placed at calculated positions in the riding-model approximation (C—H = 0.93 Å, O—H = 0.82 Å) with U_iso_(H) = 1.2U_eq_(C) or 1.5U_eq_(O). Water H atoms were located in difference Fourier maps and refined with U_iso_ = 1.5_eq_(O), and distance restrains of O—H = 0.85 (2) and H···H = 1.39 (1) Å.

### Crystal data

**Table:**

[Cu(C_8_H_5_O_4_)(NO_3_)(C_18_H_10_N_4_)(H_2_O)]	*Z* = 2
*M**_r_* = 590.99	*F*(000) = 602
Triclinic, *P*1	*D*_x_ = 1.693 Mg m^−^^3^
Hall symbol: -P 1	Mo *K*α radiation, λ = 0.71073 Å
*a* = 7.8965 (17) Å	Cell parameters from 2056 reflections
*b* = 11.295 (4) Å	θ = 3.0–23.0°
*c* = 14.533 (6) Å	µ = 1.01 mm^−^^1^
α = 112.73 (3)°	*T* = 293 K
β = 90.94 (3)°	Block, green
γ = 102.60 (2)°	0.37 × 0.32 × 0.24 mm
*V* = 1159.4 (6) Å^3^	

### Data collection

**Table:**

Bruker APEX area-detector diffractometer	5180 independent reflections
Radiation source: fine-focus sealed tube	3353 reflections with *I* > 2σ(*I*)
graphite	*R*_int_ = 0.036
φ and ω scans	θ_max_ = 27.5°, θ_min_ = 2.0°
Absorption correction: multi-scan (SADABS; Sheldrick, 1996)	*h* = −1→10
*T*_min_ = 0.707, *T*_max_ = 0.797	*k* = −13→13
6320 measured reflections	*l* = −18→18

### Refinement

**Table:**

Refinement on *F*^2^	Primary atom site location: structure-invariant direct methods
Least-squares matrix: full	Secondary atom site location: difference Fourier map
*R*[*F*^2^ > 2σ(*F*^2^)] = 0.059	Hydrogen site location: inferred from neighbouring sites
*wR*(*F*^2^) = 0.198	H atoms treated by a mixture of independent and constrained refinement
*S* = 1.02	*w* = 1/[σ^2^(*F*_o_^2^) + (0.1211*P*)^2^] where *P* = (*F*_o_^2^ + 2*F*_c_^2^)/3
5180 reflections	(Δ/σ)_max_ < 0.001
367 parameters	Δρ_max_ = 0.60 e Å^−^^3^
39 restraints	Δρ_min_ = −0.92 e Å^−^^3^

### Special details

**Table:**

Geometry. All e.s.d.'s (except the e.s.d. in the dihedral angle between two l.s. planes) are estimated using the full covariance matrix. The cell e.s.d.'s are taken into account individually in the estimation of e.s.d.'s in distances, angles and torsion angles; correlations between e.s.d.'s in cell parameters are only used when they are defined by crystal symmetry. An approximate (isotropic) treatment of cell e.s.d.'s is used for estimating e.s.d.'s involving l.s. planes.
Refinement. Refinement of *F*^2^ against ALL reflections. The weighted *R*-factor *wR* and goodness of fit *S* are based on *F*^2^, conventional *R*-factors *R* are based on *F*, with *F* set to zero for negative *F*^2^. The threshold expression of *F*^2^ > σ(*F*^2^) is used only for calculating *R*-factors(gt) *etc*. and is not relevant to the choice of reflections for refinement. *R*-factors based on *F*^2^ are statistically about twice as large as those based on *F*, and *R*- factors based on ALL data will be even larger.

### Fractional atomic coordinates and isotropic or equivalent isotropic displacement parameters (Å^2^)

**Table:**

	*x*	*y*	*z*	*U*_iso_*/*U*_eq_	
Cu1	−0.27167 (8)	0.70493 (5)	0.50884 (4)	0.0396 (2)	
C1	−0.2797 (7)	0.4150 (4)	0.4401 (3)	0.0445 (11)	
H1A	−0.2211	0.4354	0.5023	0.053*	
C2	−0.3193 (7)	0.2855 (5)	0.3703 (4)	0.0497 (13)	
H2A	−0.2915	0.2195	0.3865	0.060*	
C3	−0.4002 (7)	0.2552 (5)	0.2769 (4)	0.0428 (11)	
H3A	−0.4269	0.1684	0.2293	0.051*	
C4	−0.4426 (6)	0.3552 (4)	0.2532 (3)	0.0345 (9)	
C5	−0.5234 (6)	0.3340 (4)	0.1558 (3)	0.0342 (9)	
C6	−0.6258 (6)	0.1988 (4)	−0.0066 (3)	0.0373 (10)	
C7	−0.6553 (7)	0.0742 (4)	−0.0887 (3)	0.0432 (11)	
H7A	−0.6148	0.0059	−0.0826	0.052*	
C8	−0.7424 (7)	0.0553 (5)	−0.1756 (4)	0.0495 (12)	
H8A	−0.7629	−0.0272	−0.2287	0.059*	
C9	−0.8031 (7)	0.1561 (5)	−0.1884 (3)	0.0484 (12)	
H9A	−0.8635	0.1398	−0.2492	0.058*	
C10	−0.7739 (7)	0.2774 (5)	−0.1122 (4)	0.0485 (12)	
H10A	−0.8133	0.3445	−0.1211	0.058*	
C11	−0.6829 (6)	0.3022 (4)	−0.0187 (3)	0.0385 (10)	
C12	−0.5768 (6)	0.4395 (4)	0.1423 (3)	0.0345 (9)	
C13	−0.5442 (6)	0.5688 (4)	0.2256 (3)	0.0370 (10)	
C14	−0.6011 (7)	0.6754 (5)	0.2202 (4)	0.0449 (11)	
H14A	−0.6635	0.6660	0.1619	0.054*	
C15	−0.5634 (7)	0.7937 (5)	0.3021 (4)	0.0491 (12)	
H15A	−0.6028	0.8646	0.3003	0.059*	
C16	−0.4675 (7)	0.8076 (4)	0.3869 (4)	0.0469 (12)	
H16A	−0.4391	0.8897	0.4408	0.056*	
C17	−0.4550 (6)	0.5890 (4)	0.3143 (3)	0.0345 (9)	
C18	−0.4036 (6)	0.4815 (4)	0.3297 (3)	0.0336 (9)	
C19	0.1568 (7)	0.8271 (5)	1.0427 (4)	0.0454 (11)	
C20	0.0882 (6)	0.7361 (4)	0.9360 (3)	0.0374 (10)	
C21	0.0985 (7)	0.6051 (5)	0.9002 (4)	0.0456 (11)	
H21A	0.1474	0.5744	0.9426	0.055*	
C22	0.0371 (7)	0.5205 (5)	0.8029 (4)	0.0486 (12)	
H22A	0.0477	0.4335	0.7788	0.058*	
C23	−0.0412 (7)	0.5655 (5)	0.7404 (4)	0.0441 (11)	
H23A	−0.0831	0.5081	0.6744	0.053*	
C24	−0.0572 (6)	0.6956 (4)	0.7756 (3)	0.0352 (9)	
C25	0.0095 (6)	0.7805 (4)	0.8736 (3)	0.0364 (9)	
H25A	0.0013	0.8680	0.8978	0.044*	
C26	−0.1412 (6)	0.7470 (4)	0.7111 (3)	0.0370 (10)	
N1	−0.3230 (5)	0.5117 (4)	0.4209 (3)	0.0382 (8)	
N2	−0.4136 (5)	0.7078 (3)	0.3947 (3)	0.0390 (8)	
N3	−0.5463 (5)	0.2151 (3)	0.0821 (3)	0.0372 (8)	
N4	−0.6549 (5)	0.4244 (4)	0.0569 (3)	0.0389 (8)	
N5	0.0836 (6)	0.8341 (4)	0.4567 (3)	0.0484 (10)	
O1	0.2191 (6)	0.7958 (4)	1.1021 (3)	0.0698 (12)	
O2	0.1382 (6)	0.9481 (3)	1.0633 (2)	0.0592 (10)	
H2B	0.1490	0.9896	1.1243	0.089*	
O3	−0.1920 (5)	0.6681 (3)	0.6212 (2)	0.0463 (8)	
O4	−0.1588 (5)	0.8630 (3)	0.7480 (2)	0.0508 (9)	
O5	0.2236 (7)	0.8977 (5)	0.5054 (4)	0.0954 (17)	
O6	0.0214 (9)	0.8751 (6)	0.4026 (5)	0.113 (2)	
O7	0.0063 (5)	0.7317 (4)	0.4652 (4)	0.0729 (12)	
O1W	−0.2595 (6)	0.8908 (3)	0.5905 (2)	0.0525 (9)	
H1WA	−0.211 (8)	0.953 (4)	0.574 (4)	0.079*	
H1WB	−0.250 (8)	0.915 (5)	0.6533 (16)	0.079*	

### Atomic displacement parameters (Å^2^)

**Table:**

	*U*^11^	*U*^22^	*U*^33^	*U*^12^	*U*^13^	*U*^23^
Cu1	0.0579 (4)	0.0285 (3)	0.0284 (3)	0.0089 (2)	0.0000 (2)	0.0082 (2)
C1	0.061 (3)	0.037 (2)	0.034 (2)	0.014 (2)	−0.003 (2)	0.0122 (19)
C2	0.077 (4)	0.036 (2)	0.037 (2)	0.022 (2)	−0.004 (2)	0.012 (2)
C3	0.053 (3)	0.035 (2)	0.040 (2)	0.018 (2)	−0.002 (2)	0.0102 (19)
C4	0.038 (2)	0.030 (2)	0.036 (2)	0.0091 (18)	0.0024 (18)	0.0132 (17)
C5	0.038 (2)	0.033 (2)	0.031 (2)	0.0103 (18)	0.0010 (17)	0.0112 (17)
C6	0.039 (2)	0.037 (2)	0.034 (2)	0.0111 (19)	0.0034 (18)	0.0112 (18)
C7	0.054 (3)	0.035 (2)	0.036 (2)	0.014 (2)	0.000 (2)	0.0080 (19)
C8	0.061 (3)	0.043 (3)	0.036 (2)	0.016 (2)	−0.004 (2)	0.004 (2)
C9	0.055 (3)	0.052 (3)	0.030 (2)	0.012 (2)	−0.009 (2)	0.009 (2)
C10	0.061 (3)	0.046 (3)	0.039 (2)	0.014 (2)	−0.006 (2)	0.017 (2)
C11	0.045 (3)	0.039 (2)	0.028 (2)	0.006 (2)	−0.0012 (18)	0.0119 (18)
C12	0.040 (2)	0.032 (2)	0.032 (2)	0.0112 (18)	0.0012 (18)	0.0120 (17)
C13	0.043 (3)	0.029 (2)	0.039 (2)	0.0076 (18)	0.0063 (19)	0.0141 (18)
C14	0.058 (3)	0.039 (2)	0.040 (2)	0.017 (2)	−0.002 (2)	0.016 (2)
C15	0.070 (3)	0.033 (2)	0.048 (3)	0.022 (2)	0.001 (2)	0.014 (2)
C16	0.060 (3)	0.031 (2)	0.043 (3)	0.013 (2)	−0.006 (2)	0.0077 (19)
C17	0.043 (3)	0.027 (2)	0.033 (2)	0.0094 (18)	0.0053 (18)	0.0111 (17)
C18	0.039 (2)	0.032 (2)	0.031 (2)	0.0107 (18)	0.0029 (17)	0.0120 (17)
C19	0.058 (3)	0.044 (3)	0.038 (2)	0.022 (2)	0.007 (2)	0.016 (2)
C20	0.045 (3)	0.036 (2)	0.034 (2)	0.0164 (19)	0.0066 (19)	0.0137 (18)
C21	0.058 (3)	0.042 (2)	0.043 (3)	0.023 (2)	0.006 (2)	0.017 (2)
C22	0.064 (3)	0.036 (2)	0.044 (3)	0.020 (2)	0.005 (2)	0.009 (2)
C23	0.053 (3)	0.036 (2)	0.038 (2)	0.013 (2)	0.006 (2)	0.0075 (19)
C24	0.042 (2)	0.032 (2)	0.030 (2)	0.0073 (18)	0.0031 (18)	0.0116 (17)
C25	0.045 (3)	0.031 (2)	0.032 (2)	0.0123 (18)	0.0029 (18)	0.0100 (17)
C26	0.047 (3)	0.032 (2)	0.030 (2)	0.0065 (19)	0.0021 (18)	0.0119 (17)
N1	0.050 (2)	0.0337 (18)	0.0276 (17)	0.0102 (16)	−0.0008 (16)	0.0085 (15)
N2	0.049 (2)	0.0276 (17)	0.0358 (19)	0.0090 (16)	0.0033 (17)	0.0078 (15)
N3	0.045 (2)	0.0322 (18)	0.0342 (19)	0.0120 (16)	0.0000 (16)	0.0114 (15)
N4	0.047 (2)	0.0344 (19)	0.0336 (19)	0.0096 (16)	0.0002 (16)	0.0124 (15)
N5	0.054 (3)	0.041 (2)	0.057 (3)	0.015 (2)	0.009 (2)	0.023 (2)
O1	0.104 (3)	0.067 (3)	0.047 (2)	0.045 (2)	−0.011 (2)	0.0204 (19)
O2	0.101 (3)	0.0427 (19)	0.0304 (17)	0.024 (2)	−0.0060 (18)	0.0078 (15)
O3	0.074 (2)	0.0329 (16)	0.0273 (15)	0.0122 (16)	−0.0032 (15)	0.0083 (12)
O4	0.085 (3)	0.0340 (16)	0.0269 (15)	0.0175 (17)	−0.0052 (16)	0.0045 (13)
O5	0.079 (3)	0.071 (3)	0.118 (4)	−0.008 (3)	−0.017 (3)	0.035 (3)
O6	0.140 (5)	0.120 (5)	0.116 (5)	0.035 (4)	−0.009 (4)	0.086 (4)
O7	0.062 (3)	0.056 (2)	0.117 (4)	0.015 (2)	0.021 (2)	0.052 (3)
O1W	0.090 (3)	0.0293 (16)	0.0361 (17)	0.0128 (17)	−0.0001 (18)	0.0122 (14)

### Geometric parameters (Å, °)

**Table:**

Cu1—O1W	1.952 (3)	C14—C15	1.370 (7)
Cu1—O3	1.960 (3)	C14—H14A	0.9300
Cu1—N1	2.000 (4)	C15—C16	1.373 (7)
Cu1—N2	2.001 (4)	C15—H15A	0.9300
Cu1—O7	2.284 (4)	C16—N2	1.331 (6)
C1—N1	1.336 (6)	C16—H16A	0.9300
C1—C2	1.381 (6)	C17—N2	1.363 (5)
C1—H1A	0.9300	C17—C18	1.454 (6)
C2—C3	1.374 (6)	C18—N1	1.343 (5)
C2—H2A	0.9300	C19—O1	1.188 (6)
C3—C4	1.403 (6)	C19—O2	1.324 (6)
C3—H3A	0.9300	C19—C20	1.501 (7)
C4—C18	1.392 (6)	C20—C21	1.387 (6)
C4—C5	1.452 (6)	C20—C25	1.389 (6)
C5—N3	1.327 (5)	C21—C22	1.371 (7)
C5—C12	1.426 (6)	C21—H21A	0.9300
C6—N3	1.352 (5)	C22—C23	1.394 (7)
C6—C11	1.409 (6)	C22—H22A	0.9300
C6—C7	1.418 (6)	C23—C24	1.393 (6)
C7—C8	1.346 (7)	C23—H23A	0.9300
C7—H7A	0.9300	C24—C25	1.387 (6)
C8—C9	1.400 (7)	C24—C26	1.491 (6)
C8—H8A	0.9300	C25—H25A	0.9300
C9—C10	1.355 (7)	C26—O4	1.250 (5)
C9—H9A	0.9300	C26—O3	1.261 (5)
C10—C11	1.425 (6)	N5—O6	1.201 (6)
C10—H10A	0.9300	N5—O5	1.215 (6)
C11—N4	1.358 (5)	N5—O7	1.236 (5)
C12—N4	1.310 (6)	O2—H2B	0.8200
C12—C13	1.458 (6)	O1W—H1WA	0.841 (19)
C13—C17	1.372 (6)	O1W—H1WB	0.842 (19)
C13—C14	1.402 (6)		
			
O1W—Cu1—O3	94.58 (14)	C14—C15—H15A	120.0
O1W—Cu1—N1	171.25 (17)	C16—C15—H15A	120.0
O3—Cu1—N1	89.65 (14)	N2—C16—C15	122.3 (4)
O1W—Cu1—N2	92.03 (15)	N2—C16—H16A	118.9
O3—Cu1—N2	164.17 (16)	C15—C16—H16A	118.9
N1—Cu1—N2	82.02 (15)	N2—C17—C13	123.4 (4)
O1W—Cu1—O7	97.54 (17)	N2—C17—C18	115.2 (4)
O3—Cu1—O7	89.57 (16)	C13—C17—C18	121.4 (4)
N1—Cu1—O7	90.14 (16)	N1—C18—C4	123.9 (4)
N2—Cu1—O7	103.82 (17)	N1—C18—C17	116.1 (4)
N1—C1—C2	122.1 (4)	C4—C18—C17	120.0 (4)
N1—C1—H1A	119.0	O1—C19—O2	123.7 (5)
C2—C1—H1A	119.0	O1—C19—C20	125.0 (5)
C3—C2—C1	119.4 (4)	O2—C19—C20	111.3 (4)
C3—C2—H2A	120.3	C21—C20—C25	119.6 (4)
C1—C2—H2A	120.3	C21—C20—C19	119.7 (4)
C2—C3—C4	120.0 (4)	C25—C20—C19	120.7 (4)
C2—C3—H3A	120.0	C22—C21—C20	120.6 (5)
C4—C3—H3A	120.0	C22—C21—H21A	119.7
C18—C4—C3	116.3 (4)	C20—C21—H21A	119.7
C18—C4—C5	119.5 (4)	C21—C22—C23	119.6 (4)
C3—C4—C5	124.2 (4)	C21—C22—H22A	120.2
N3—C5—C12	122.1 (4)	C23—C22—H22A	120.2
N3—C5—C4	118.1 (4)	C24—C23—C22	120.7 (4)
C12—C5—C4	119.8 (4)	C24—C23—H23A	119.6
N3—C6—C11	121.4 (4)	C22—C23—H23A	119.6
N3—C6—C7	119.3 (4)	C25—C24—C23	118.7 (4)
C11—C6—C7	119.2 (4)	C25—C24—C26	119.1 (4)
C8—C7—C6	119.6 (4)	C23—C24—C26	122.2 (4)
C8—C7—H7A	120.2	C24—C25—C20	120.7 (4)
C6—C7—H7A	120.2	C24—C25—H25A	119.6
C7—C8—C9	122.0 (4)	C20—C25—H25A	119.6
C7—C8—H8A	119.0	O4—C26—O3	123.8 (4)
C9—C8—H8A	119.0	O4—C26—C24	119.4 (4)
C10—C9—C8	120.1 (4)	O3—C26—C24	116.8 (4)
C10—C9—H9A	119.9	C1—N1—C18	118.3 (4)
C8—C9—H9A	119.9	C1—N1—Cu1	128.4 (3)
C9—C10—C11	120.1 (5)	C18—N1—Cu1	113.1 (3)
C9—C10—H10A	120.0	C16—N2—C17	117.7 (4)
C11—C10—H10A	120.0	C16—N2—Cu1	129.4 (3)
N4—C11—C6	121.7 (4)	C17—N2—Cu1	112.9 (3)
N4—C11—C10	119.3 (4)	C5—N3—C6	116.1 (4)
C6—C11—C10	119.0 (4)	C12—N4—C11	116.4 (4)
N4—C12—C5	122.1 (4)	O6—N5—O5	118.2 (5)
N4—C12—C13	118.4 (4)	O6—N5—O7	121.5 (5)
C5—C12—C13	119.5 (4)	O5—N5—O7	120.3 (5)
C17—C13—C14	117.5 (4)	C19—O2—H2B	109.5
C17—C13—C12	119.5 (4)	C26—O3—Cu1	128.9 (3)
C14—C13—C12	123.0 (4)	N5—O7—Cu1	121.6 (3)
C15—C14—C13	119.0 (4)	Cu1—O1W—H1WA	123 (4)
C15—C14—H14A	120.5	Cu1—O1W—H1WB	117 (4)
C13—C14—H14A	120.5	H1WA—O1W—H1WB	111 (3)
C14—C15—C16	120.1 (4)		
			
N1—C1—C2—C3	2.4 (8)	C26—C24—C25—C20	179.9 (4)
C1—C2—C3—C4	−0.3 (8)	C21—C20—C25—C24	−0.8 (7)
C2—C3—C4—C18	−2.2 (7)	C19—C20—C25—C24	−178.7 (4)
C2—C3—C4—C5	178.2 (5)	C25—C24—C26—O4	−4.0 (7)
C18—C4—C5—N3	174.7 (4)	C23—C24—C26—O4	176.9 (5)
C3—C4—C5—N3	−5.6 (7)	C25—C24—C26—O3	176.6 (4)
C18—C4—C5—C12	−5.8 (6)	C23—C24—C26—O3	−2.5 (7)
C3—C4—C5—C12	173.9 (4)	C2—C1—N1—C18	−1.7 (7)
N3—C6—C7—C8	176.6 (5)	C2—C1—N1—Cu1	−177.8 (4)
C11—C6—C7—C8	−2.3 (7)	C4—C18—N1—C1	−1.0 (7)
C6—C7—C8—C9	1.1 (8)	C17—C18—N1—C1	177.3 (4)
C7—C8—C9—C10	0.4 (9)	C4—C18—N1—Cu1	175.7 (4)
C8—C9—C10—C11	−0.6 (8)	C17—C18—N1—Cu1	−6.1 (5)
N3—C6—C11—N4	2.5 (7)	O3—Cu1—N1—C1	−10.2 (4)
C7—C6—C11—N4	−178.7 (4)	N2—Cu1—N1—C1	−176.7 (4)
N3—C6—C11—C10	−176.8 (4)	O7—Cu1—N1—C1	79.3 (4)
C7—C6—C11—C10	2.0 (7)	O3—Cu1—N1—C18	173.5 (3)
C9—C10—C11—N4	−179.9 (5)	N2—Cu1—N1—C18	7.0 (3)
C9—C10—C11—C6	−0.6 (8)	O7—Cu1—N1—C18	−96.9 (3)
N3—C5—C12—N4	1.5 (7)	C15—C16—N2—C17	0.8 (8)
C4—C5—C12—N4	−178.0 (4)	C15—C16—N2—Cu1	179.2 (4)
N3—C5—C12—C13	−178.5 (4)	C13—C17—N2—C16	2.0 (7)
C4—C5—C12—C13	2.0 (6)	C18—C17—N2—C16	−175.8 (4)
N4—C12—C13—C17	−176.8 (4)	C13—C17—N2—Cu1	−176.7 (4)
C5—C12—C13—C17	3.1 (7)	C18—C17—N2—Cu1	5.5 (5)
N4—C12—C13—C14	3.7 (7)	O1W—Cu1—N2—C16	1.2 (5)
C5—C12—C13—C14	−176.3 (4)	O3—Cu1—N2—C16	115.8 (6)
C17—C13—C14—C15	0.9 (7)	N1—Cu1—N2—C16	174.7 (5)
C12—C13—C14—C15	−179.6 (5)	O7—Cu1—N2—C16	−97.1 (5)
C13—C14—C15—C16	1.6 (8)	O1W—Cu1—N2—C17	179.7 (3)
C14—C15—C16—N2	−2.6 (9)	O3—Cu1—N2—C17	−65.7 (6)
C14—C13—C17—N2	−2.8 (7)	N1—Cu1—N2—C17	−6.8 (3)
C12—C13—C17—N2	177.7 (4)	O7—Cu1—N2—C17	81.4 (3)
C14—C13—C17—C18	174.9 (4)	C12—C5—N3—C6	−1.0 (6)
C12—C13—C17—C18	−4.6 (7)	C4—C5—N3—C6	178.5 (4)
C3—C4—C18—N1	2.9 (7)	C11—C6—N3—C5	−0.9 (6)
C5—C4—C18—N1	−177.4 (4)	C7—C6—N3—C5	−179.7 (4)
C3—C4—C18—C17	−175.3 (4)	C5—C12—N4—C11	0.1 (7)
C5—C4—C18—C17	4.4 (6)	C13—C12—N4—C11	−179.9 (4)
N2—C17—C18—N1	0.4 (6)	C6—C11—N4—C12	−2.0 (7)
C13—C17—C18—N1	−177.5 (4)	C10—C11—N4—C12	177.3 (4)
N2—C17—C18—C4	178.7 (4)	O4—C26—O3—Cu1	11.6 (7)
C13—C17—C18—C4	0.8 (7)	C24—C26—O3—Cu1	−169.0 (3)
O1—C19—C20—C21	−1.2 (8)	O1W—Cu1—O3—C26	−4.4 (4)
O2—C19—C20—C21	179.2 (5)	N1—Cu1—O3—C26	−176.7 (4)
O1—C19—C20—C25	176.7 (5)	N2—Cu1—O3—C26	−118.7 (6)
O2—C19—C20—C25	−2.9 (7)	O7—Cu1—O3—C26	93.1 (4)
C25—C20—C21—C22	2.3 (8)	O6—N5—O7—Cu1	−54.7 (7)
C19—C20—C21—C22	−179.8 (5)	O5—N5—O7—Cu1	123.5 (5)
C20—C21—C22—C23	−2.0 (8)	O1W—Cu1—O7—N5	−30.2 (5)
C21—C22—C23—C24	0.2 (8)	O3—Cu1—O7—N5	−124.7 (4)
C22—C23—C24—C25	1.3 (7)	N1—Cu1—O7—N5	145.6 (4)
C22—C23—C24—C26	−179.6 (5)	N2—Cu1—O7—N5	63.8 (5)
C23—C24—C25—C20	−1.0 (7)		

### Hydrogen-bond geometry (Å, °)

**Table:**

*D*—H···*A*	*D*—H	H···*A*	*D*···*A*	*D*—H···*A*
O1W—H1WA···O6^i^	0.84 (2)	2.08 (3)	2.856 (7)	153 (5)
O1W—H1WA···O5^i^	0.84 (2)	2.41 (3)	3.165 (6)	151 (6)
O1W—H1WB···O4	0.84 (2)	1.88 (4)	2.565 (5)	138 (6)
O2—H2B···O4^ii^	0.82	1.94	2.722 (4)	159

Symmetry codes: (i) −*x*, −*y*+2, −*z*+1; (ii) −*x*, −*y*+2, −*z*+2.
